# Erythrasma in 4 skin of color patients with hidradenitis suppurativa

**DOI:** 10.1016/j.jdcr.2021.05.013

**Published:** 2021-06-02

**Authors:** Diana Gruenstein, Jacob Oren Levitt

**Affiliations:** Department of Dermatology, Icahn School of Medicine at Mount Sinai, New York, New York

**Keywords:** *Corynebacterium minutissimum*, cutaneous infection, erythrasma, hidradenitis suppurativainfection, intertrigo, skin occlusion, skin of color, Wood's lamp, HS, hidradenitis suppurativa

## Introduction

Erythrasma is a superficial cutaneous infection caused by *Corynebacterium minutissimum* that typically presents as intertriginous erythematous scaly plaques or patches. Skin occlusion and increased moisture predispose to erythrasma.^1^ We report 4 cases of erythrasma in patients with hidradenitis suppurativa (HS).

## Case 1

**Fig 1:**
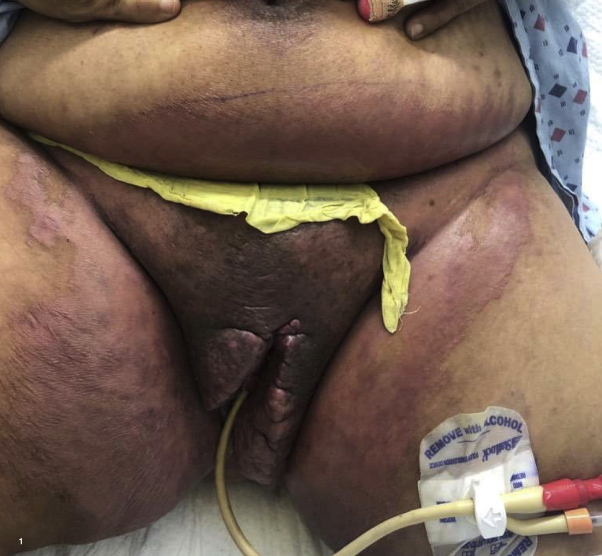
Erythrasma of the lower abdomen and medial aspect of thighs in a hospitalized preoperative patient with chronic hidradenitis suppurativa.

A 41-year-old Latina woman with severe HS for more than a decade presented with a flare of her vulva, perianal, and perineal regions. The patient was a smoker and had a disease of the axillae, abdomen, groin, and labia treated with infliximab (treatment with adalimumab having failed), spironolactone, dapsone, intralesional triamcinolone, and aggressive surgical intervention of her axillae and panniculectomy. Physical examination revealed extensive erythematous, edematous plaques involving the vulva, consistent with chronic HS. Deep fissures with a gelatinous pseudomembrane extended along the inguinal folds. Erythematous, scaly patches of the lower abdomen and medial thighs were also noted (Fig 1), consistent with erythrasma. Wood's lamp revealed coral-red fluorescence of the lower abdomen and medial aspect of thighs. The erythrasma resolved with topical application of clindamycin, twice daily.

## Case 2

**Fig 2:**
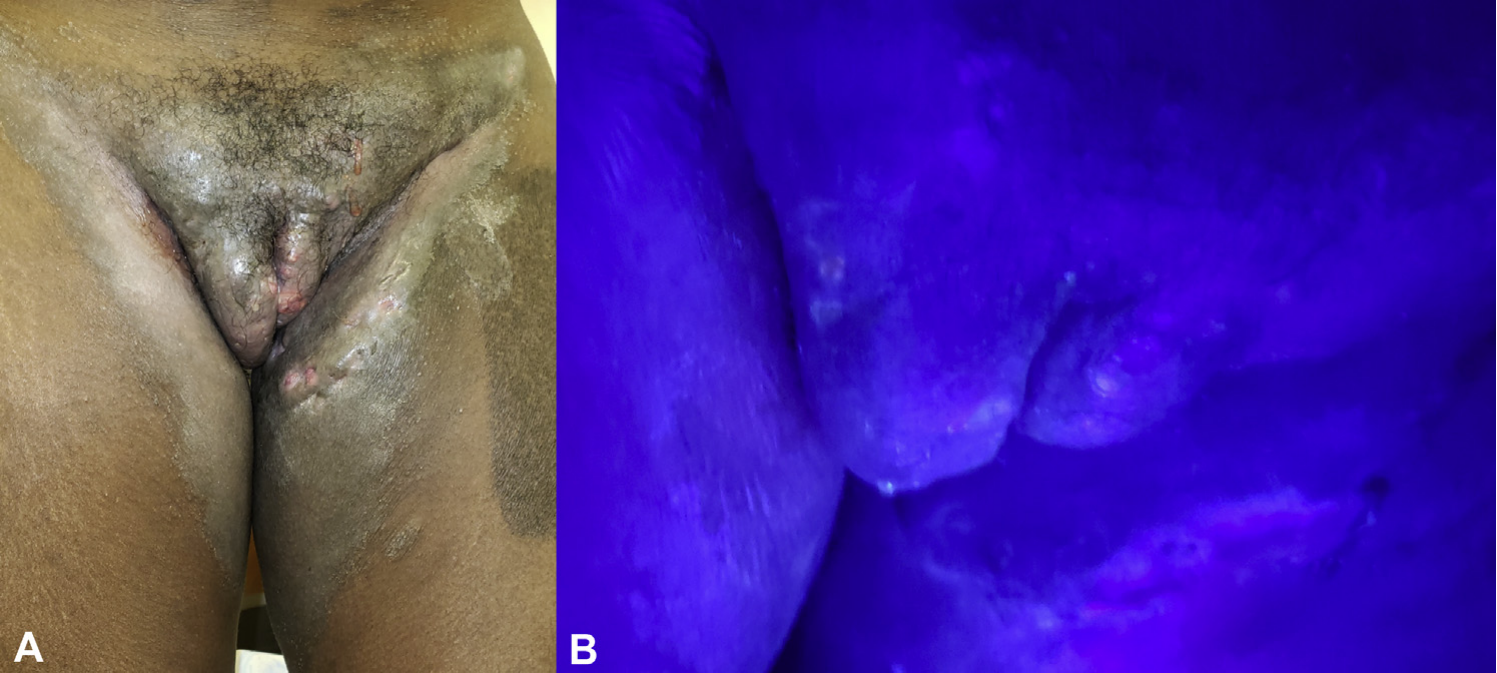
Erythrasma and active hidradenitis suppurativa. **A,** Natural light. **B,** Coral-red fluorescence under Wood's lamp.

A 20-year-old Black woman with HS since 3 years presented with an active disease of her suprapubic area, labia majora, and medial aspect of the left thigh. The patient had a disease of the axillae, buttocks, and breasts treated with aggressive surgery, doxycycline, chlorhexidine wash, topical clindamycin, intralesional triamcinolone, and adalimumab. Physical examination revealed draining sinus tracts in the left thigh, suprapubic area, and labia majora with granulating ulcers of the sacrum and right buttock. Erythematous, scaly plaques were noted on the medial aspect of the left thigh (Fig 2, *A*). Wood's lamp revealed coral-red fluorescence (Fig 2, *B*) of the medial aspect of the left thigh, consistent with erythrasma. The erythrasma responded to oral erythromycin base 500 mg that was administered 4 times daily for 7 days, after failing a course of topical clindamycin and benzoyl peroxide combination gel.

## Case 3

**Fig 3:**
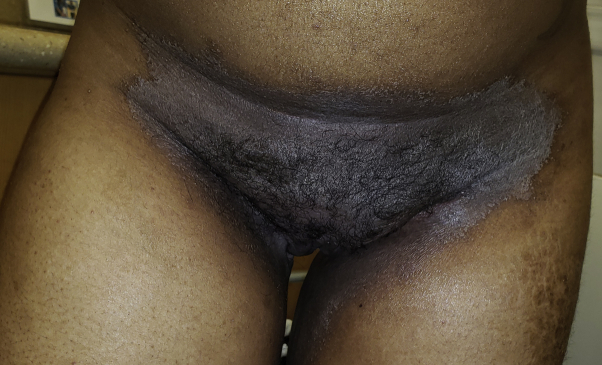
Erythrasma of the lower abdomen and inguinal folds in a patient with erosive hidradenitis suppurativa.

A 23-year-old Black woman presented for a follow-up of severe HS of many years. The patient had a disease of the axillae, inguinal folds, abdomen, labia majora, and perineum that had been managed with secukinumab (after failing treatment with infliximab and adalimumab), intralesional triamcinolone, and aggressive surgical debridement. Physical examination revealed ulcers of bilateral inguinal creases (consistent with erosive HS) and well-defined erythematous scaly plaques of the lower abdomen and inguinal folds (Fig 3), consistent with erythrasma. Wood's lamp revealed coral-red fluorescence. The erythrasma responded to clindamycin 1% lotion, applied twice daily for 1 month.

## Case 4

**Fig 4:**
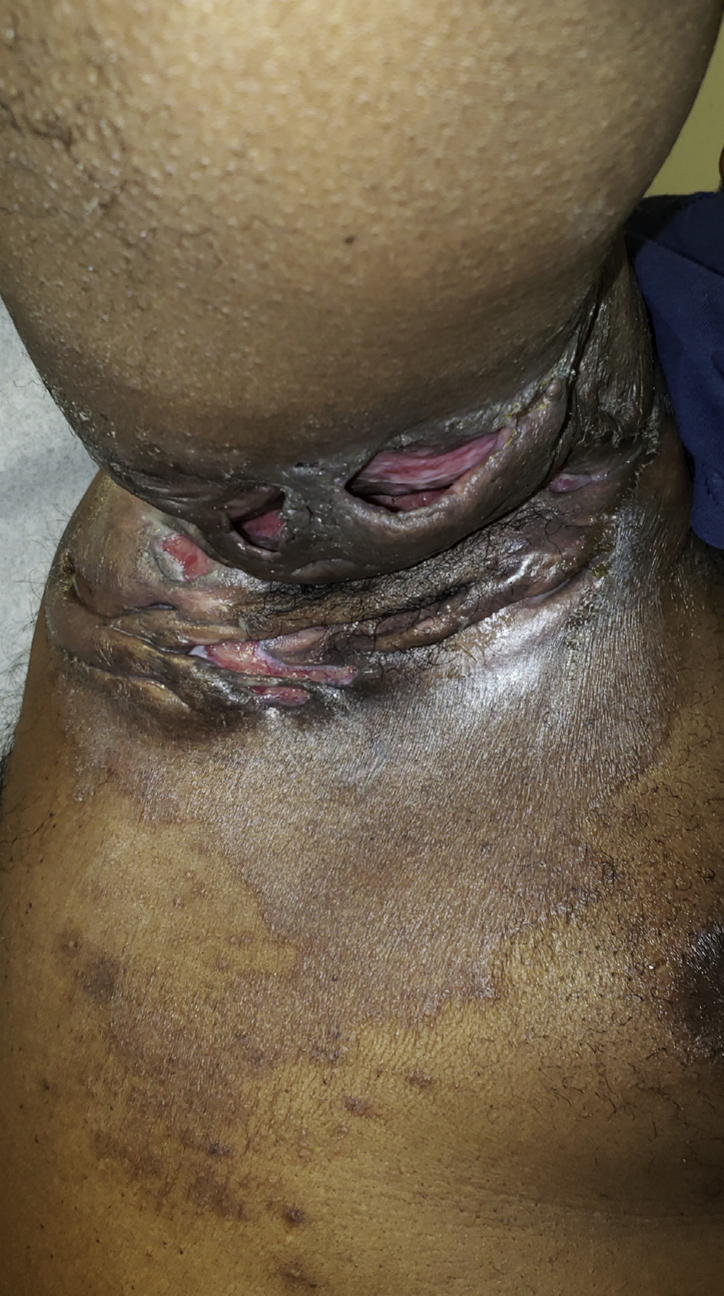
Erythrasma of the axillae in a patient with chronic hidradenitis suppurativa.

A 37-year-old Black man presented for consultation of HS of many years. The patient had a disease of the groin and axillae that had been managed with adalimumab and surgical debridement. Physical examination revealed multiple draining sinuses and nodules in the groin and axillae, and erythematous scaly plaques of the axillae (Fig 4). Wood's lamp was performed for the axillae, revealing coral-red fluorescence.

## Discussion

The presence of intertriginous erythematous scaly plaques in patients with HS is often overlooked as tinea cruris, candidiasis, inverse psoriasis, or simply dismissed as intertrigo from maceration. HS commonly affects intertriginous areas and presents with macerated patches overlying the draining ulcers, making the distinction between active HS and erythrasma difficult.

To our knowledge, the association between HS and erythrasma has not been studied, though it is intuitive. Patients with diabetes mellitus, obesity, immunosuppression, and skin conditions that are prone to skin occlusion and moisture are at an increased risk of developing erythrasma.^1^ Similarly, HS is associated with immune dysregulation and obesity and predisposes to bacterial colonization and disruption of the skin barrier. Cutaneous skin infections are more common in patients with HS.^2^

Our series suggests that the clinical suspicion of erythrasma should remain high when patients with HS present with intertriginous involvement. Although erythrasma might have a little clinical impact on HS, leaving erythrasma untreated can result in patient discomfort, malodor, and dyspigmentation.^1^ We encourage the use of Wood's lamp examination for intertriginous erythematous plaques seen in patients with HS and treatment of any coincident erythrasma.

## Conflicts of interest

Dr Levitt has served on advisory boards for Corona Psoriasis Registry, NACE, UCB, Leo Pharma, Novartis, Helsinn, AbbVie, and Arcutis Biotherapeutics and has been a consultant for Novartis and AbbVie. Author Gruenstein has no conflicts of interest to declare.
